# NEWS-2 Accuracy in Predicting Mortality and Severe Morbidity Among Hospitalized COVID-19 Patients: A Prospective Cohort Study

**DOI:** 10.3390/jcm13216558

**Published:** 2024-10-31

**Authors:** Mahdi Tarabeih, Jamal Qaddumi, Islam Mohammad Tukhi, Wasef Na’amnih

**Affiliations:** 1 Nephrology Department, An-Najah National University Hospital, Nablus P450, Palestinian Territory; mahdita@mta.ac.il; 2 Public Health Department, Faculty of Medicine and Health Sciences, An-Najah National University, Nablus P450, Palestinian Territory; jamal9877@najah.edu; 3 Rafedia Surgical Governmental Hospital, Palestinian Ministry of Health, Nablus P450, Palestinian Territory; islamtukhi@gmail.com

**Keywords:** NEWS-2, COVID-19, sensitivity, predictive

## Abstract

**Background**: Early risk stratification tools for COVID-19 patients have been indicated yet there are few data about their ability to effectively detect clinical deterioration among COVID-19 patients. **Objectives**: To evaluate the NEWS-2 to predict severe morbidity and mortality for COVID-19 patients admitted to hospitals. **Methods**: We conducted a prospective cohort study among adult COVID-19 patients with a confirmed diagnosis who were admitted to the inpatient units at COVID-19 Martyrs Medical Military Complex Hospital, from 1 March 2022, until 29 February 2023. NEWS-2 scores were measured at admission and 6, 12, 24, and 48 h after their admission to the hospital using receiver operating characteristic (ROC) curves. **Results**: Overall, 192 adult COVID-19 patients aged 25–94 years (mean = 62.1, SD = 13.9) were enrolled. Of those, 49.0% were males, 47.4% were vaccinated, and 53.6% had diabetes. The 192 enrolled patients were classified into NEWS-2 score categories, with almost 13% (12.5%) falling into the high-risk category already upon admission. The mean NEWS-2 scores were excellent predictors of mechanical ventilation, admission to the ICU, and mortality, as indicated by an AUROC of 0.94 (95% CI: 0.88–1.00, *p* < 0.001), 0.91 (95% CI: 0.87–0.96, *p* < 0.001), and 0.96 (95% CI: 0.92–1.00, *p* < 0.001), respectively. Significant differences in mean NEWS-2 scores were found between the participating patients, both with and without comorbidity in the course of the patient’s stay in the ICU, and mortality (*p* = 0.004, *p* = 0.043, respectively). Positive correlations of the high NEWS-2 scores were revealed using a multiple linear regression model, indicating the necessity of administering non-invasive ventilatory assistance (*p* = 0.013), hospitalization for a minimum of six days (*p* = 0.013), and admission to the ICU (*p* = 0.006). Nonetheless, there was a negative association between mortality and the NEWS-2 score (*p* < 0.001). **Conclusions**: The NEWS-2 had moderate sensitivity and specificity in predicting the deterioration of patients with COVID-19 whereas there was high sensitivity and specificity in predicting the mortality for patients with COVID-19, both with and without comorbidity. Our findings support the utility of NEWS-2 monitoring as a sensitive approach for initially assessing COVID-19 patients. It could be helpful to enhance the accuracy of predictive performance by supplementing the score parameters by adding biological parameters in addition to clinical judgment.

## 1. Introduction

COVID-19 cases have been registered in Wuhan, China since December 2019. Due to the global spread and severity of the cases, the WHO identified the situation as a COVID-19 pandemic in March 2020 [1]. The universal scoring system known as National Early Warning Score-2 (NEWS-2) was formulated to enhance safety for patients suffering from hypercapnic respiratory failure by proposing the use of a separate scoring system for the oxygen saturation (SpO2) parameter and to monitor the risk of patient deterioration. This is because COVID-19 has a variety of clinical presentations, from asymptomatic transmission to becoming life-threatening [2,3,4]. In 2012, the Royal College of Physicians recommended the National Early Warning Score (NEWS) as a standardized early warning system to assess illness severity, identify the risk of patient deterioration, and support clinical judgment [5]. The features of NEWS are itemized in William’s study [6]. Five years later, in December 2017, the Royal College of Physicians instituted a revised, improved version of NEWS-2 to be used as the standardized early warning system and authorized its operation in ambulance services and acute care hospitals [3]. By 2020, when COVID-19 infections started their global spread, NEWS-2 was already in place and was being implemented to evaluate baseline illness severity and to track clinical deterioration. It was shown to accurately predict clinical deterioration risk in acutely ill patients suffering from sepsis or undifferentiated disease [6]. Accordingly, NEWS-2 scores were expected to be higher in patients with acute COVID-19-related pneumonia, which presents chiefly with severe hypoxia, a need for oxygen therapy, an elevated respiratory rate, fever, tachycardia, and, in rarer cases, mental confusion [7]. The NEWS-2 Calculation Application is a digital tool that has facilitated the calculation and tracking of NEWS-2 scores in clinical practice to identify the early deterioration of patients; thus, it is important in conjunction with clinical judgment and interpreting the NEWS-2 score in the context of the patient’s clinical condition. Standardizing how the NEWS-2 score is calculated and interpreted can bring about improved patient outcomes and may save lives [3,7].

Clinical characteristics of the patient, including elevated respiratory rate, fever, and low systolic blood pressure, may mean they require respiratory support such as non-invasive pressure support, invasive ventilation, or admission to an intensive care unit (ICU). This has placed an added burden on the capacity and workflow of emergency departments and ICUs in hospitals globally [8,9]. Therefore, identifying the disease severity for these patients early is important as failure to recognize a patient’s deteriorating condition in a hospital setting may result in life-threatening conditions, a lengthened hospital stay, and significant disabling consequences that may lead to unanticipated hospital admissions or readmissions, increasing hospital morbidity and mortality [10]. NEWS-2 seems to be a robust predictor of COVID-19 inpatient hospital deaths [11,12,13,14,15]. This is extremely important because it validates the ability of NEWS-2 to support clinical judgment while also providing a standardized communication tool in a short time frame, taking into account the limited resources and operational demands that hospitals faced during the COVID-19 pandemic outbreak in the emergency phase [14]. Consequently, patients facing clinical deterioration or at risk of deterioration would receive an initial assessment on time. NEWS-2 is associated with reduced mortality rates and deferred medical interventions, however, at times numerical scores caused overestimating at the expense of holistic clinical analysis and the situational context [3,16,17].

NEWS-2 has a critical role in monitoring patients who are at risk of clinical deterioration in ICU settings. It measures physiological parameters such as respiration rate, heart rate, oxygen saturation, and consciousness level and integrates these to predict the chances of adverse outcomes such as admission to an ICU, the necessity to use mechanical ventilation, and mortality. The NEWS-2 scoring system has been highly effective in guiding medical decisions on employing such respiratory interventions as low-flow oxygen, a high-flow nasal cannula (HFNC), and mechanical ventilation [18,19]. Patients on low-flow oxygen typically have lower NEWS-2 scores, indicating that their clinical status is less severe. Low-flow O_2_ therapy is generally used for stable patients or those who do not need more aggressive respiratory intervention. However, if a patient’s condition worsens, reflected by an increasing NEWS-2 score, healthcare providers may escalate treatment. Low-flow O_2_ is used in the ICU for stable patients or those who do not require more aggressive respiratory support. Monitoring using NEWS-2 helps assess the necessity of escalating oxygen therapy [18]. HFNC is a non-invasive therapy used to deliver oxygen at high flow rates, helping patients who do not respond adequately to low-flow oxygen. In clinical trials with HFNC, it was suggested that in certain patient populations, it may prevent the necessity for mechanical ventilation, especially for patients who suffer from acute respiratory failure [18,19]. HFNC was shown to have successful outcomes among COVID-19 patients in managing acute hypoxemic respiratory failure with a relatively low intubation rate (10.8%) and a low mortality rate (2.7%) [18]. This indicates that HFNC can be highly effective in reducing the escalation of respiratory support when used in the initial stages of respiratory disorder as identified by a rising NEWS-2 score.

Patients ventilated due to severe respiratory failure are typically those with the highest NEWS-2 scores, pointing to severe illness. Despite its life-saving capabilities, mechanical ventilation has been correlated with longer ICU hospitalization and higher mortality rates. Patient’s failure to respond to HFNC treatment or declining to the need for mechanical ventilation of long duration and a hospital stay of six days or more correlated significantly with the severity of illness and worse outcomes; this worsening is signaled by a higher NEWS-2 score [18].

NEWS-2 is a clinical scoring system used in hospitals to classify those at risk for clinical decline. The score is calculated using six physiological parameters on a scale of 0 to 20, signaling the level of risk and guiding the level of monitoring and intervention. The score is classified according to three levels of risk that indicate different monitoring protocols, of special importance in ICU units: (1) low-risk category (NEWS-2 score 0–4): low-risk patients who require observation every 4 to 6 h; (2) medium-risk category (NEWS-2 score 5–6): increase in monitoring to at least every hour for medium-risk patients who may require increased medical support or even l ICU admission; (3) high-risk category (NEWS-2 score ≥ 7): high-risk patients are monitored continuously or every 15–30 min at least. A high-risk score is a powerful predictor of severe clinical deterioration, which might indicate the need for mechanical ventilation or resuscitation [18,20].

The integration of NEWS-2 scores within the broader clinical assessment is vital, considering the myriad factors influencing patient health evaluations. Such scores should be integrated with condition-specific observations that could also necessitate heightened clinical intervention. Patient engagement and understanding are fundamental in both the assessment process and in decision-making [16,21]. The use of NEWS-2 has extended to emergency departments and pre-hospital settings, buoyed by growing evidence and experiential learning [22,23]. The National Institute for Health and Care Excellence recommends registering for these vital parameters at hospital admission or during initial evaluations with a recent audit of 156 hospitals demonstrating compliance within 30 min in 77% of cases [16,24]. Escalation thresholds signal the need for more intensive clinical evaluation to respond to severe morbidity, risk of clinical decline, or change in patient status. Accordingly, we explored the prognostic accuracy of NEWS-2 in predicting severe morbidity and mortality for hospitalized COVID-19 patients.

The contribution of our study is unique in using the NEWS-2 score for predicting outcomes for COVID-19 patients. In contrast to previous studies, it had the following advantages: (1) where previous studies were retrospective, ours was designed as a prospective cohort study and used a prospective methodology. It measured NEWS-2 scores at multiple time points (beginning with admission, and then at 6 h, 12 h, 24 h, and 48 h post-admission). Most of the previous studies only evaluated NEWS-2 scores during admission to the emergency room or upon admission to the hospital [25,26,27]. Our methodology produces a more detailed analysis of how NEWS-2 scores develop in predicting the progression of patient illness, whereby the assessment of NEWS-2 scores at several time points from the onset of COVID-19 infection enhances both the reliability and the validity (sensitivity and specificity) of the statistical data. (2) The population in our study is unique because it included patients from two large hospitals located in the Palestinian Authority, where limited resources and operational constraints, among others, created certain challenges to the healthcare system during the COVID-19 pandemic. This geographic and healthcare dimension has had scant scholarly attention so our study offers a new viewpoint on the applicability of the NEWS-2 score in populations characterized by low socioeconomic status and insufficient resources; this reinforces the validity and reliability of the tool. (3) The focus of our study is the predictive accurateness of the NEWS-2 scores for specific severe illness and mortality outcomes, including the need for mechanical ventilation, ICU admission, and mortality, in contrast to earlier studies that measured the use of NEWS-2 in general [7,28,29]. (4) In addition, we provide ROC curve analyses to assess NEWS-2’s performance during several periods which provides an added understanding of its prognostic accurateness; among them were analyses dividing the research participants into groups by sex, vaccination, and comorbidity. (5) Insofar as we have seen, this is the first study conducted in the Palestinian Authority that assesses NEWS-2’s prognostic accuracy specifically for COVID-19 at the pandemic’s peak. Thus, we believe that the study contributes new insights and information to local clinical practices that promote an appreciation of the NEWS-2 tool in varied healthcare settings characterized by limited resources and challenging operating constraints.

## 2. Materials and Methods

### 2.1. Study Design and Population

A prospective cohort study was performed among adult patients who were admitted to the inpatient units of COVID-19 at the Martyrs Medical Military Complex Hospital; this also functioned as a certified testing and treatment facility for COVID-19 located at Nablus, Palestinian Authority from 1 March 2022 to 28 February 2023.

### 2.2. Inclusion and Exclusion Criteria

Following the Royal College of Physicians’ 2017 standards, we included adult patients (≥18 years of age) with a positive nasal and/or oropharyngeal PCR swab of COVID-19. Patients under the age of 18 years, pregnant women, patients suffering from spinal cord injuries, and patients readmitted to the ICU during the research period were excluded from participation in the research.

### 2.3. Collection of Data and Clinical Definitions

The patient’s physiological measurements including temperature, heart rate, respiratory rate, systolic blood pressure, saturation, consciousness degree, and additional oxygen required were collected; these measurements were used as the full inputs for the NEWS-2 calculation from admission until discharge (dead/alive). We defined severe morbidity by one of the following events: initiation of respiratory support (low flow oxygen, high flow nasal cannula, non-invasive positive pressure ventilation (NIPPV), or invasive mechanical ventilation), at the ICU admission, or death in the hospital. When none of the above occurred, observations were documented at discharge.

The NEWS-2 severity score is divided into groups: an aggregate score of 1–4 is considered low risk and the ward nurse must then assess the patient promptly within six hours to determine whether to increase the monitoring frequency or to escalate clinical treatment. A NEWS-2 value of 5–6 for any single parameter constitutes a moderate risk and requires that the patient be evaluated every hour with a follow-up by another physician or nurse to determine the reason and to modify the monitoring frequency or to escalate clinical treatment. A score of 7 and above indicates that the patient will likely be transferred to a higher dependency care ward after the nurse notifies the attending physician that the patient requires urgent evaluation by the critical care team. We evaluated the performance of the NEWS-2 scoring system as predictive of morbidity and mortality at five specified points in time which were: at admission, 6 h after admission, and then again 12, 24, and 48 h after hospitalization. We calculated the mean NEWS-2 score for the scores obtained at all the points in time at admission and during hospitalization. For each point in time, we used the NEWS-2 score to provide a holistic approach where NEWS-2 can intervene in predicting morbidity and mortality. It is noteworthy that the patient’s clinical condition was taken into account when the score was evaluated and the parameters were set and calculated manually. We then verified the results with the NEWS-2 calculator, a support resource for SIGN guidelines. The NEWS-2 value could be rapidly calculated from seven easily available measures, making it an essential part of a reliable clinical evaluation. The NEWS-2 Calculation Application is a digital tool that facilitates the monitoring and calculation of NEWS-2 scores in clinical practice practically by the early identification of patients in the first stages of clinical deterioration. It is important to combine the use of NEWS-2 with clinical judgment and to interpret the score with the severity of the patient’s clinical condition. A standardized system of calculation and interpretation of the NEWS-2 scores can bring about improved patient outcomes and lowered mortality rates.

### 2.4. Data Analysis

Receiver operating characteristic (ROC) curves were used to examine NEWS-2’s ability in predicting outcomes, however, logistic and linear regression were used to pool determining cut-off points by the Youden index for the variables measured, likelihood ratios, sensitivity, and specificity. ANOVA tests were conducted to assess how the implementation affected various outcomes. Significant variables with a two-sided *p* value < 0.05 in the Student’s *t*-test were included in the multiple linear regression model using SPSS version 28 (IBM, Armonk, NY, USA).

### 2.5. Ethics Approval

Ethics approval for this study was carried out following the Declaration of Helsinki. The Institutional Review Board approved the study protocol with the number 2022–25 in March 2022. All procedures were performed in accordance with the local regulations and guidelines of An-Najah National University Hospital. The participants signed an informed consent form, agreeing to participate in the research and authorizing the publication of its findings.

## 3. Results

Overall, 192 adult COVID-19 patients aged 25–94 years (mean = 62.1, SD = 13.9) were enrolled. Of those, 49.0% were males, 47.4% were vaccinated, and 53.6%, 10.4%, and 5.7% had diabetes, chronic kidney disease, and cancer, respectively.

Table 1 shows the classification of patients into NEWS-2 score categories as low (monitored every 4–6 h), medium (monitored every 1 h), and high risk (monitored continuously) based on their NEWS-2 scores measured at admission and at the other time intervals (6, 12, 24, and 48 h) after their admission to the hospital. Of the 192 patients, 112 (58.3%) were classified as low risk, 56 (29.2%) were classified as medium risk, and 24 (12.5%) were classified as high risk at admission. An increase was observed in the probability of low-risk NEWS-2 score categories during a hospital stay: 6 h (59.4%), 12 h (65.6%), 24 h (71.4%), and 48 h (80.2%) after admission vs. the admission score (58.3%) compared to the decrease in the percentage of medium-risk NEWS-2 score measured at the different points in time from hospital admission (29.2%) and up to 48 h after admission (8.3%). However, the percentage of high-risk NEWS-2 scores increased slightly during hospitalization at up to 12 h (17.2%) compared to the score at admission (12.5%), while the percentages measured at 24 and 48 h were 14.1% and 11.5%, respectively (Table 1).

**Figure 1:**
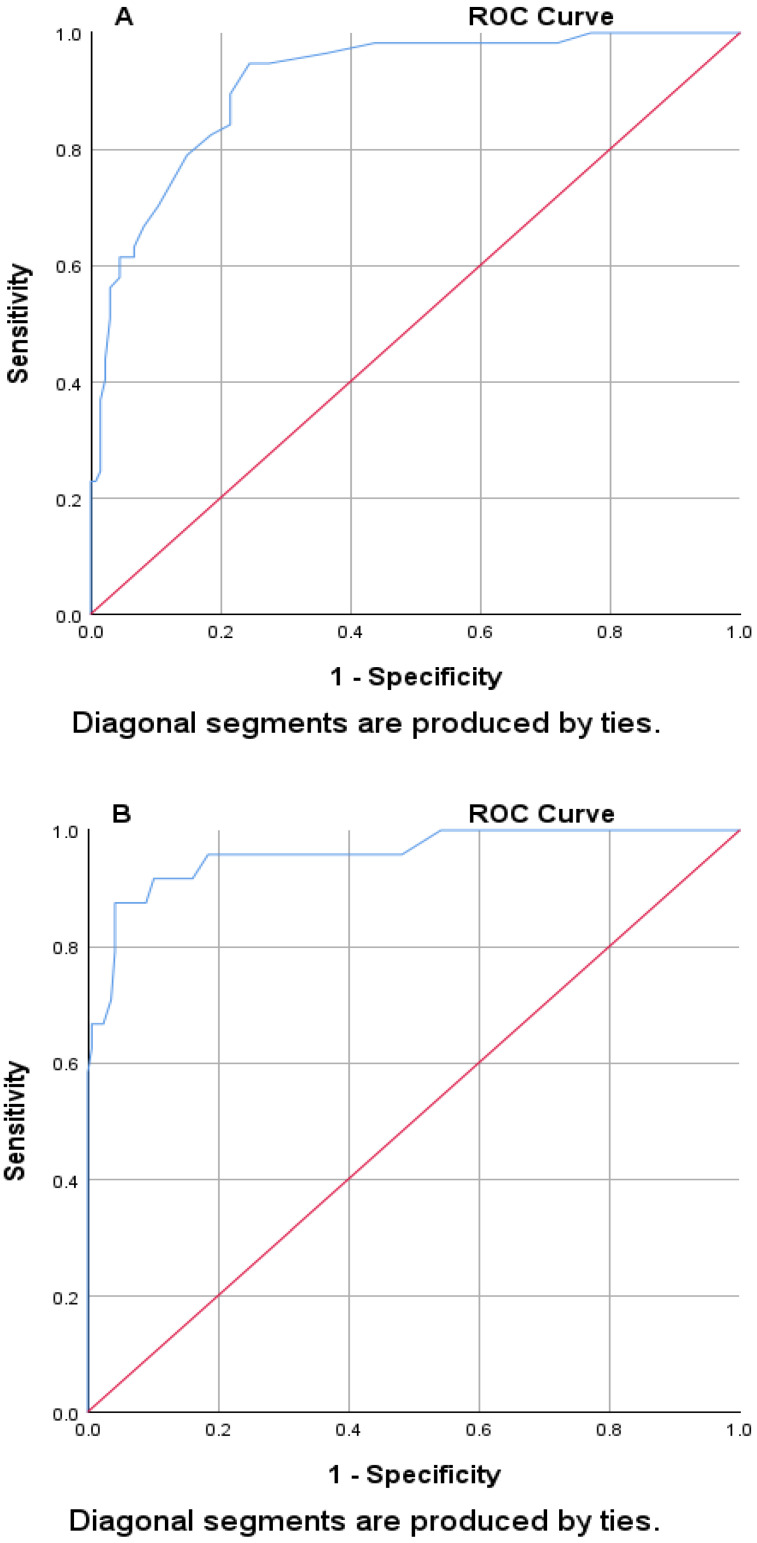

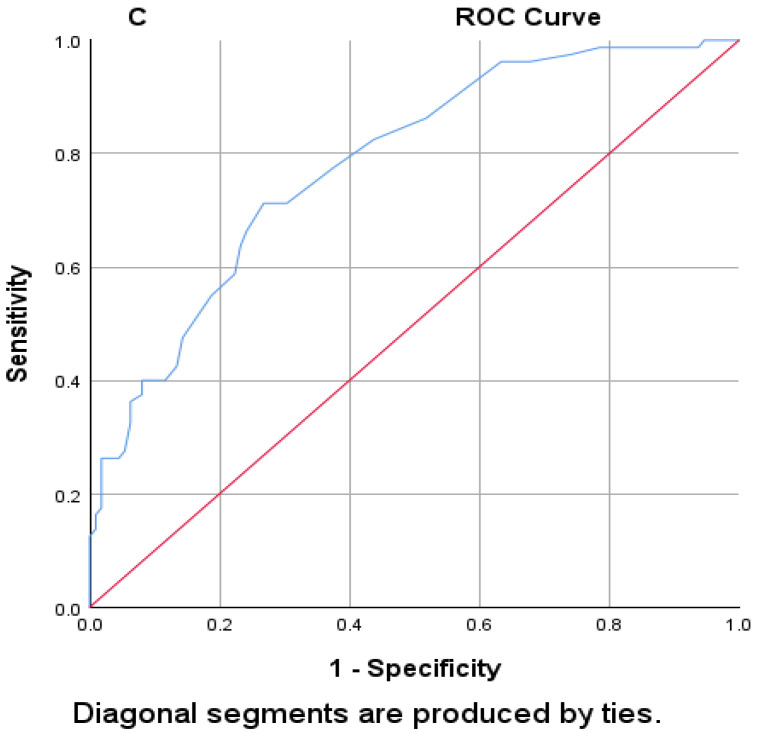
ROC curve using mean NEWS-2 scores for (**A**) patients admitted to the ICU; (**B**) mortality; and (**C**) patient’s hospitalization stay for ≥six days. The red line expresses the reference line; however, the blue line expresses the mean NEWS-2.

Table 2 and Figure 1 indicated AUROC, optimal cut-off, sensitivity, and 1-specificity values of all the NEWS-2 scores. The mean NEWS-2 score calculated during the patient’s hospitalization predicted the need for treatment by low-flow O_2_ fairly well as indicated by an AUROC of 0.74 (95% CI: 0.64–0.84, *p* < 0.001), with a cutoff point of 3.7 that accurately predicted 52.7% of low-flow O_2_ cases with a specificity of 81.0%. However, the mean NEWS-2 scores during the patient’s hospitalization were good and excellent predictors of the need for high-flow O_2_ and mechanical ventilation, respectively, as indicated by an AUROC of 0.87 (95% CI: 0.82–0.92, *p* < 0.001) and 0.94 (95% CI: 0.88–1.00, *p* < 0.001), respectively, with cutoff points of 5.0 and 6.3 that accurately predict 56.1% and 88.2% of the high-flow O_2_ and mechanical ventilation, respectively, with a specificity of 90.4% and 89.7%. The mean NEWS-2 score during the patient’s hospitalization term proved to be an excellent predictor of the need for administering NIPPV as indicated by an AUROC of 0.95 (95% CI: 0.90–0.99, *p* < 0.001), with a cutoff point of 5.7 that predicted accurately 92.0% of the NIPPV cases with a specificity of 90.4% (Table 2).

The ROC curve analyses revealed that the average NEWS-2 scores during the patient’s term of hospitalization were excellent predictors of admission to the ICU (Figure 1A) and of mortality (Figure 1B) as shown by an AUROC of 0.91 (95% CI: 0.87–0.96, *p* < 0.001) and 0.96 (95% CI: 0.92–1.00, *p* < 0.001), respectively, with cutoff points of 4.7 and 5.9 that could correctly predict 70.2% and 87.5% of ICU admissions and mortality with specificity of 89.6% and 91.9%, respectively. However, the mean NEWS-2 score during the patient’s hospitalization term predicted a hospital stay of six or more days fairly well, which was indicated by an AUROC of 0.78 (95% CI: 0.72–0.85, *p* < 0.001), with a cutoff point of 4.5 that accurately predicted 55.0% of the cases with a specificity of 81.2% (Table 2) (Figure 1C).

We conducted ROC curve analyses also to predict outcomes by sex, vaccination, and comorbidities. We found no significant differences between males and females on the mean NEWS-2 scores, the AUROC values, and the cutoff points during the patient’s six-day or more hospital stay, admission to ICU, and mortality (*p* = 0.397, *p* = 0.380, *p* = 0.441, respectively) (Supplementary Figures S1A–S3A). Nor did we find significant differences between vaccinated and non-vaccinated patients on the mean NEWS-2 scores, the AUROC values, and the cutoff points during the six-day or more hospital stay, admission to ICU, and mortality (*p* = 0.545, *p* = 0.718, *p* = 0.153, respectively) (Supplementary Figures S1B–S3B). However, we did find significant differences between participants with comorbidity and patients without comorbidities during the patient’s hospital stay at admission to the ICU and mortality (*p* = 0.004, *p* = 0.043, respectively) as indicated by an AUROC of 0.896 (95% CI: 0.86–0.94) and 0.987 (95% CI: 0.91–0.99) for participants with and without comorbidity, respectively, with cutoff points of 3.9 that predicted accurately 92.0% of the cases with a specificity of 73.8% for participants with comorbidity and 4.3 that accurately predicted 100.0% of the cases with a specificity of 97.4% for participants without comorbidity at admission to the ICU (Supplementary Figure S2C). Regarding mortality, the AUROC values were 0.971 (95% CI: 0.88–0.99) and 1.000 (95% CI: 1.00–1.12) for participants with and without comorbidity, respectively, with cutoff points of 6.5 that predict accurately 91.0% of the cases with a specificity of 94.6% for participants with comorbidity and 7.1 that accurately predicted 100.0% of the cases with a specificity of 100.0% for participants without comorbidity (Supplementary Figure S3C).

The significant predictors of high and low NEWS-2 scores were identified by using a multiple linear regression model. Table 3 shows positive correlations of the high NEWS-2 scores with the necessity of NIPPV treatment (*p* = 0.013), a hospital stay of six or more days (*p* = 0.013), and admission to the ICU (*p* = 0.006). However, mortality was negatively correlated with the NEWS-2 score (*p* < 0.001). No significant correlations were found between age, comorbidities (diabetes, hypertension, chronic liver disease, cardiovascular disease), and the NEWS-2 score (Table 3).

## 4. Discussion

In our study, we explored the prognostic accuracy of NEWS-2 to predict severe morbidity and mortality in hospitalization for COVID-19 patients. In terms of predicting the combined outcome of the requirement for intense respiratory assistance, ICU admission, or in-hospital mortality, the NEWS-2 commonly displays good discrimination. Our study findings revealed various levels of predictive accuracy, as follows: the mean NEWS-2 scores were a fair predictor of the need for low-flow O_2_ and a good predictor of the need for high-flow O_2_, whereas it proved to be an excellent predictor of the need for mechanical ventilation during the patient’s hospital stay. These findings were consistent with a 2020 study that indicated a good prognostic performance in oxygen saturation levels for predicting death in patients with COVID-19 infection [30]. Thus, the NEWS-2 showed high sensitivity and discrimination with the advantage gained from combining a specific scale for patients with hypercapnic respiratory failure. The high sensitivity of NEWS-2 supports its use as an effective tool for accurately assessing COVID-19 patients upon hospital admission. The cutoff point of high-flow O_2_ to predict severe morbidity and mortality in hospital settings for COVID-19 patients participating in our study was similar to the cutoff point that was reported for severe or critical COVID-19 patients who were treated with HFNC in the ICU of the Shanghai Public Health Clinical Center (cutoff point of 4.45) [31].

We also found that the average NEWS-2 scores during the patient’s hospitalization predicted a six-day or more hospitalization period fairly well and had high accuracy in predicting ICU admission and mortality as indicated by an AUROC. These findings aligned with several other small cohort studies that examined the predictive performance of baseline NEWS-2 along with other clinical scoring systems used for predicting clinical outcomes for COVID-19 patients that relied on one single measurement taken upon admission to the hospital. The baseline NEWS-2 had a better prediction of mortality than CURB-65 (0.85 (0.81–0.89)) and performed better than qSOFA (0.73 (0.69–0.78)) with an AUROC of 0.81 (95% CI 0.77–0.85) in admission of 654 COVID-19 hospitalized patients in China [32]. In a study conducted in Korea, NEWS-2 ≥ 5 predicted an AUROC of 0.98 but a positive value of 0.59 for a future event [33]. However, the original NEWS-2 expected values for ICU admission and mortality were AUROC of 0.92 (95% CI 0.84–1.00) and 0.76 (0.62–0.90) for qSOFA, respectively.

The original NEWS-2 that predicted a combined negative outcome of admission to the ICU or mortality had an AUROC of 0.79 (95% CI 0.66–0.91) in 66 hospitalized Norwegian patients with qSOFA’s AUROC of 0.62 (0.45–0.81) and CURB-65’s AUROC of 0.58 (0.41–0.76) [11]. The predictive value of NEWS-2 for admission to the ICU was similar with an AUROC of 0.90 (CI, 0.82–0.97) in 68 patients with severe COVID-19 [34,35]. “Serious event” for hospitalized patients was defined when one of the following occurred: death, unexpected shift to ICU, or beginning non-invasive ventilation, with equivalent AUROC scores for predicting serious events of 0.837 (0.748–0.943) for admission using NEWS-2 and 0.846 (0.735–0.939) for admission using NEWS-2 stratified by age [13,34].

The main advantages of our study are: a relatively large sample size, as we included all COVID-19 patients admitted from the beginning of the epidemic including all components of the NEWS-2 score; the NEWS-2 scores were measured at admission and 6, 12, 24, and 48 h after their admission to the hospital, and it is the first study in the Palestinian Authority to examine the prognostic accuracy of NEWS-2 to predict severe morbidity and mortality among hospitalized COVID-19 patients. However, our study had some limitations: patients with serious illness might have been more severely ill already at admission; a piece of data concerning biological parameters was missing that could help improve predictive performance by supplementing the score parameters with biological parameters (e.g., laboratory blood tests and the like) apart from the clinical judgment, and the absence of cutoff points in the discussion limits the ability to translate AUROC values into actionable clinical thresholds as well as more reliable interpretations and applications of the NEWS-2 scores. For example, the reported AUROC for ICU admissions and mortality may be compelling, but cutoff values of scores are also required for clinicians to accurately judge when intervention is necessary.

## 5. Conclusions

The mean NEWS-2 scores registered for patients during their period of hospitalization were excellent predictors of admission to the ICU and mortality with a moderate sensitivity and specificity in predicting severe morbidity and mortality in hospitalization for COVID-19 patients and a high sensitivity and specificity for predicting the mortality for COVID-19 patients both with comorbidity and without. Our findings support the utility of NEWS-2 monitoring as a sensitive approach for initially assessing COVID-19 patients. Predictability might be improved by adding biological parameters to supplement the score parameters (for example, using laboratory blood tests and others) besides the clinical performance judgment. Our findings can contribute to a better understanding of the benefits of using NEWS-2 in patients with COVID-19, which could be useful for implementing its use in clinical practice.

## Figures and Tables

**Table 1:** Classification of patients into NEWS-2 score categories.

	NEWS-2 Score Measured Time											
	At Admission		6 h		12 h		24 h		48 h		Average NEWS-2 Score	
NEWS-2 Score Category	*n*	%	*n*	%	*n*	%	*n*	%	*n*	%	*n*	%
Low risk (monitor every 4–6 h)	112	58.3	114	59.4	126	65.6	137	71.4	154	80.2	143	74.5
Medium risk (monitor every 1 h)	56	29.2	45	23.4	33	17.2	28	14.6	16	8.3	26	13.5
High risk (monitor continuously)	24	12.5	33	17.2	33	17.2	27	14.1	22	11.5	23	12.0

**Table 2:** NEWS-2 to predict severe morbidity and mortality in hospitalization for COVID-19 patient ROC curve analyses.

Variable	95% CI							
	AUC	SE	*p*	Lower	Upper	Proposed Cutoff Point	Sensitivity	1-Specificity
Low-flow O_2_	0.74	0.05	<0.001	0.64	0.84	3.7	0.527	0.190
High-flow nasal cannula	0.87	0.03	<0.001	0.82	0.92	5.0	0.561	0.096
Mechanical ventilation	0.94	0.03	<0.001	0.88	1.00	6.3	0.882	0.103
NIPPV	0.95	0.02	<0.001	0.90	0.99	5.7	0.920	0.096
Hospital stay ≥ 6 days	0.78	0.03	<0.001	0.72	0.85	4.5	0.550	0.188
ICU	0.91	0.02	<0.001	0.87	0.96	4.7	0.702	0.104
Mortality	0.96	0.02	<0.001	0.92	1.00	5.9	0.875	0.089

ICU: Intensive care unit; NIPPV: Use of non-invasive positive-pressure ventilation.

**Table 3:** Multiple linear regression model of associations between predictors and NEWS-2 scores.

Variable *	Unstandardized Coefficients	SE	Standardized Coefficients	t	*p*
Age	0.25	0.24	0.06	1.07	0.287
Diabetes	0.14	0.25	0.03	0.58	0.565
Hypertension	−0.06	0.26	−0.01	−0.24	0.813
Chronic liver disease	−0.15	0.36	−0.02	−0.41	0.681
Cardiovascular disease	0.34	0.25	0.07	1.39	0.167
Low-flow O_2_	0.46	0.25	0.08	1.84	0.068
High-flow nasal cannula	0.29	0.33	0.06	0.90	0.368
Use of non-invasive positive-pressure ventilation	1.35	0.54	0.20	2.52	0.013
Mechanical ventilation	−0.79	0.66	−0.10	−1.20	0.231
Length of hospital stay	0.60	0.24	0.13	2.51	0.013
ICU	1.03	0.38	0.21	2.75	0.006
Mortality	−3.20	0.48	−0.46	−6.73	<0.001

ICU: Intensive care unit. * A multiple linear regression model included significant variables in the Student’s *t*-tests.

## Data Availability

All data generated or analyzed during this study are included in this article. Further inquiries can be directed to the corresponding author.

## Supplementary Materials

The following supporting information can be downloaded at: https://www.mdpi.com/article/10.3390/jcm13216558/s1.

## References

[1] Benlghazi A., Benali S., Bouhtouri Y., Belouad M., Massoudi H., Kouach J. (2021). SARS-CoV-2 infection in pregnant women; epidemiological, clinical, biological and evolutionary profile in 16 cases: The COVID-19 experience in the Moroccan Military Hospital in Benslimane. Pan Afr. Med. J..

[2] Jebril N. (2020). World Health Organization Declared a Pandemic Public Health Menace: A Systematic Review of the Coronavirus Disease 2019 “COVID-19”. https://papers.ssrn.com/sol3/papers.cfm?abstract_id=3566298.

[3] Royal College of Physicians (2017). National Early Warning Score (NEWS) 2. Standardizing the Assessment of Acute-Illness Severity in the NHS.

[4] Connell C.J., Endacott R., Cooper S. (2021). The prevalence and management of deteriorating patients in an Australian emergency department. Australas. Emerg. Care..

[5] Royal College of Physicians (2012). National Early Warning Score (NEWS): Standardizing the Assessment of Acute-Illness Severity in the NHS.

[6] Williams B. (2022). The National Early Warning Score: From concept to NHS implementation. Clin. Med..

[7] Williams B. (2022). Evaluation of the utility of NEWS2 during the COVID-19 pandemic. Clin. Med..

[8] Huang C., Wang Y., Li X., Ren L., Zhao J., Hu Y., Zhang L., Fan G., Xu J., Gu X. (2020). Clinical features of patients infected with 2019 novel coronavirus in Wuhan, China. Lancet.

[9] Zhou F., Yu T., Du R., Fan G., Liu Y., Liu Z., Xiang J., Wang Y., Song B., Gu X. (2020). Clinical course and risk factors for mortality of adult inpatients with COVID-19 in Wuhan, China: A retrospective cohort study. Lancet.

[10] Hammond N.E., Spooner A.J., Barnett A.G., Corley A., Brown P., Fraser J.F. (2013). The effect of implementing a modified early warning scoring (MEWS) system on the adequacy of vital sign documentation. Aust. Crit. Care.

[11] Myrstad M., Ihle-Hansen H., Tveita A.A., Andersen E.L., Nygård S., Tveit A., Berge T. (2020). National Early Warning Score 2 (NEWS2) on admission predicts severe disease and in-hospital mortality from COVID-19—A prospective cohort study. Scand. J. Trauma. Resusc. Emerg. Med..

[12] Sze S., Pan D., Williams C.M.L., Wong N., Sahota A., Bell D., Tang J.W., Wiselka M., Stephenson I., Pareek M. (2021). Letter to the Editor: Variability but not admission or trends in NEWS2 score predicts clinical outcome in elderly hospitalized patients with COVID-19. J. Infect..

[13] Baker K.F., Hanrath A.T., van der Loeff I.S., Kay L.J., Back J., Duncan C.J. (2021). National Early Warning Score 2 (NEWS2) to identify inpatient COVID-19 deterioration: A retrospective analysis. Clin Med..

[14] Rigoni M., Torri E., Nollo G., Delle Donne L., Cozzio S. (2021). NEWS2 is a valuable tool for appropriate clinical management of COVID-19 patients. Eur. J. Intern. Med..

[15] Robert G., Marie-Claire H., Jessie M., Darran R., Victoria R., Paul M. (2018). qSOFA, SIRS and NEWS for predicting inhospital mortality and ICU admission in emergency admissions treated as sepsis. Emerg. Med. J..

[16] Welch J., Dean J., Hartin J. (2022). Using NEWS2: An essential component of reliable clinical assessment. Clin. Med..

[17] Scott L.J., Redmond N.M., Tavaré A., Little H., Srivastava S., Pullyblank A. (2020). Association between National Early Warning Scores in primary care and clinical outcomes: An observational study in UK primary and secondary care. Br. J. Gen. Pract..

[18] Shoukri A.M. (2021). High flow nasal cannula oxygen and non-invasive mechanical ventilation in management of COVID-19 patients with acute respiratory failure: A retrospective observational study. Egypt. J. Broncho..

[19] Al-Dorzi H.M., Kress J., Arabi Y.M. (2022). High-Flow Nasal Oxygen and Noninvasive Ventilation for COVID-19. Crit. Care Clin..

[20] Hernández G., Roca O., Colinas L. (2017). High-flow nasal cannula support therapy: New insights and improving performance. Annual Update in Intensive Care and Emergency Medicine.

[21] Holland M., Kellett J. (2022). A systematic review of the discrimination and absolute mortality predicted by the National Early Warning Scores according to different cut-off values and prediction windows. Eur. J. Intern. Med..

[22] Prytherch D.R., Smith G.B., Schmidt P.E., Featherstone P.I. (2010). ViEWS–Towards a national early warning score for detecting adult inpatient deterioration. Resuscitation.

[23] Kovacs C., Jarvis S.W., Prytherch D.R., Meredith P., Schmidt P.E., Briggs J.S., Smith G.B. (2016). Comparison of the National Early Warning Score in non-elective medical and surgical patients. Br. J. Surg..

[24] National Institute for Health and Care Excellence (2007). Acutely Ill Patients in Hospital: Recognition of and Response to Acute Illness in Adults in Hospital: Clinical Guideline [CG50].

[25] Pimentel M.A.F., Redfern O.C., Malycha J., Meredith P., Prytherch D., Briggs J., Young J.D., Clifton D.A., Tarassenko L., Watkinson P.J. (2021). Detecting deteriorating patients in the hospital: Development and validation of a novel scoring system. Am. J. Respir. Crit. Care Med..

[26] Brink A., Alsma J., Verdonschot R.J.C.G., Rood P.P.M., Zietse R., Lingsma H.F., Schuit S.C.E. (2019). Predicting mortality in patients with suspected sepsis at the Emergency Department: A retrospective cohort study comparing qSOFA, SIRS, and National Early Warning Score. PLoS ONE.

[27] Jo S., Jeong T., Lee J.B., Jin Y., Yoon J., Park B. (2020). The prognostic value of platelet-to-lymphocyte ratio on in-hospital mortality in admitted adult traffic accident patients. PLoS ONE.

[28] Martín-Rodríguez F., Sanz-García A., Ortega G.J., Delgado-Benito J.F., García Villena E., Mazas Pérez-Oleaga C., Castro Villamor M.A. (2022). One-on-one comparison between qCSI and NEWS scores for mortality risk assessment in patients with COVID-19. Ann. Med..

[29] Wibisono E., Hadi U., Arfijanto M.V., Rusli M., Rahman B.E., Asmarawati T.P., Rahayu D.R.P. (2022). National Early Warning Score (NEWS) 2 predicts hospital mortality from COVID-19 patients. Ann. Med. Surg..

[30] Liu F.Y., Sun X.L., Zhang Y., Ge L., Wang J., Liang X., Li J.F., Wang C.L., Xing Z.T., Chhetri J.K. (2020). Evaluation of the risk prediction tools for patients with coronavirus disease 2019 in Wuhan, China: A single-centered, retrospective, observational study. Crit Care Med..

[31] Ai T., Zhang Z., Tan Z., Shi Z., Li H., Zhang S., Zhao X., Yao Y., Li W., Gao Y. (2023). Modified Respiratory Rate Oxygenation Index: An Early Warning Index for the Need of Intubation in COVID-19 Patients with High-Flow Nasal Cannula Therapy. J. Emerg. Med..

[32] Fan G., Tu C., Zhou F., Liu Z., Wang Y., Song B., Gu X., Wang Y., Wei Y., Li H. (2020). Comparison of severity scores for COVID-19 patients with pneumonia: A retrospective study. Eur. Respir. J..

[33] Jang J.G., Hur J., Hong K.S., Lee W., Ahn J.H. (2020). Prognostic Accuracy of the SIRS, qSOFA, and NEWS for Early Detection of Clinical Deterioration in SARS-CoV-2 Infected Patients. J. Korean Med. Sci..

[34] Martín-Rodríguez F., López-Izquierdo R., Del Pozo Vegas C., Delgado-Benito J.F., Carbajosa-Rodríguez V., Diego-Rasilla M.N., Martín-Conty J.L., Mayo-Iscar A., Otero de la Torre S., Méndez-Martín V. (2019). Accuracy of National Early Warning Score 2 (NEWS2) in Prehospital Triage on In-Hospital Early Mortality: A Multi-Center Observational Prospective Cohort Study. Prehosp Disaster Med..

[35] Aliberti M.J.R., Covinsky K.E., Garcez F.B., Smith A.K., Curiati P.K., Lee S.J., Dias M.B., Melo V.J.D., do Rego-Júnior O.F., Richinho V.P. (2021). A fuller picture of COVID-19 prognosis: The added value of vulnerability measures to predict mortality in hospitalized older adults. Age Ageing.

