# Supplementary material for: NEWS-2 Accuracy in Predicting Mortality and Severe Morbidity Among Hospitalized COVID-19 Patients: A Prospective Cohort Study
Source: J Clin Med. 2024 Oct 31;13(21):6558. doi: 10.3390/jcm13216558 (PMC11546082; doi:10.3390/jcm13216558)
Supplement: Supplementary file 1 [file jcm-13-06558-s001.zip › jcm-3197885-supplementary.pdf]

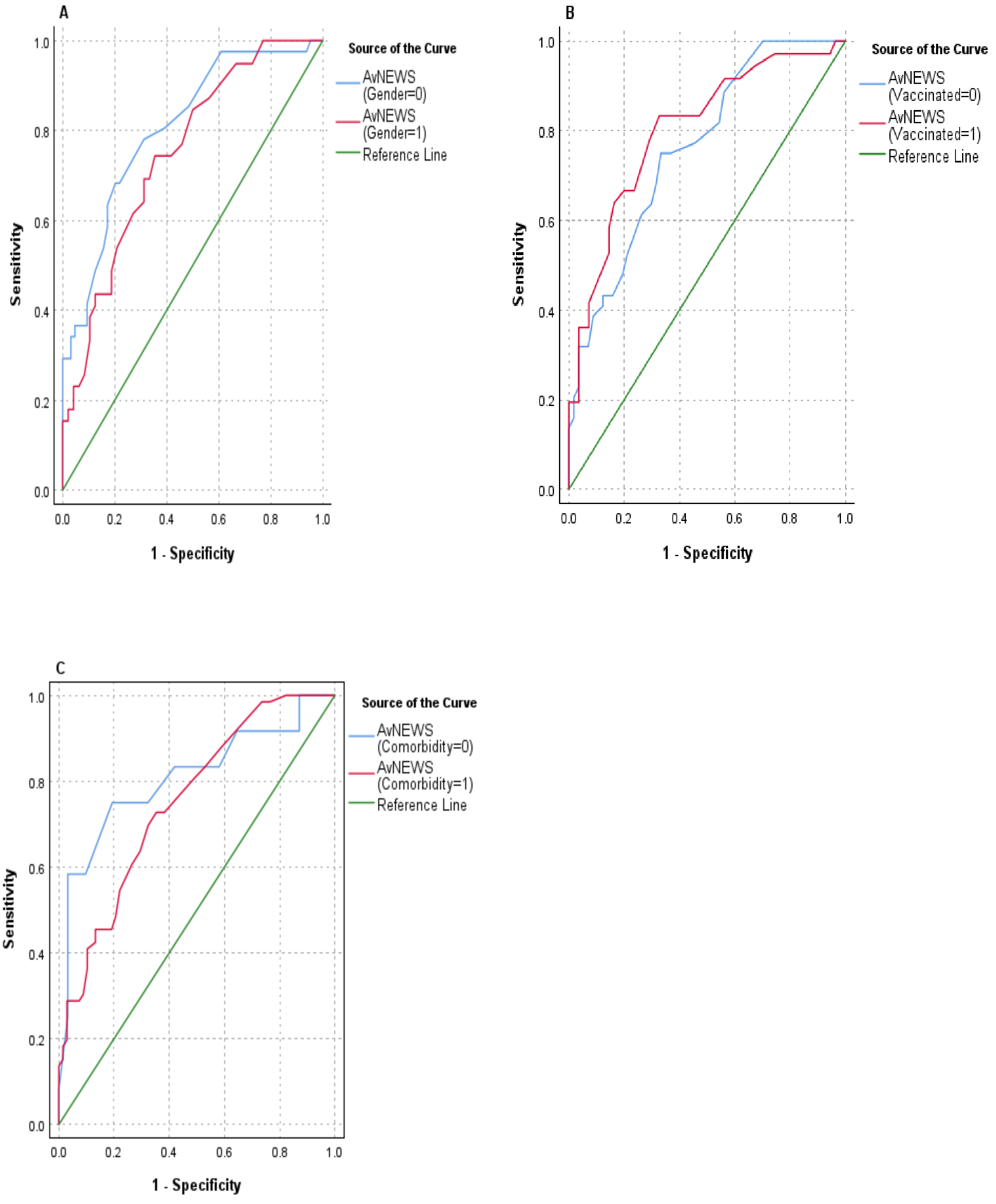

Figure 1S. ROC curve for patients staying in the hospital for 6 days or more using mean NEWS scores by **(A)** sex (Female=0, Male=1); **(B)** by received vaccine (Unvaccinated=0, Vaccinated=1); (**C**) Comorbidity (Without comorbidity=0, With comorbidity=1).

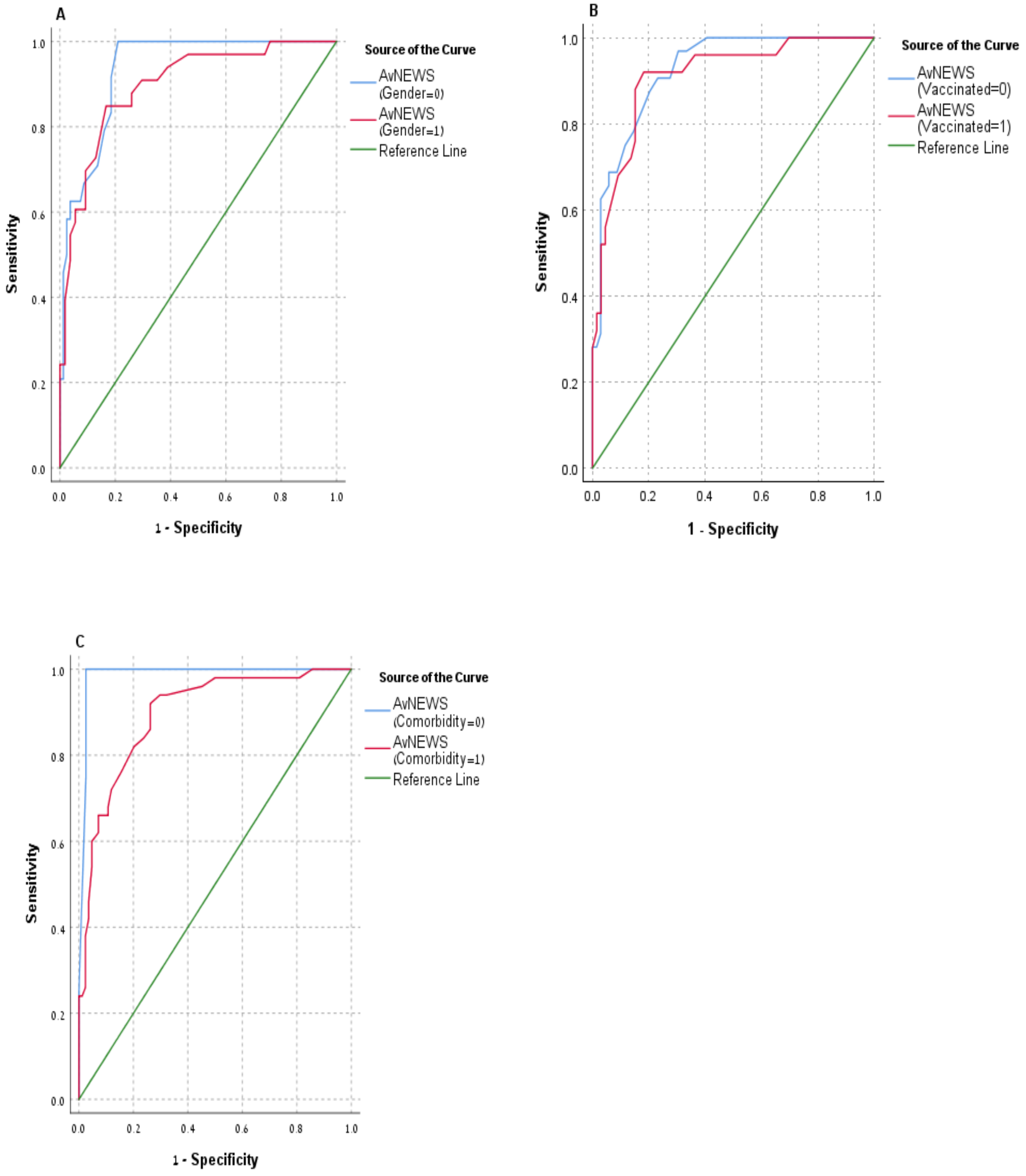

Figure 2S. ROC curve for patients admitted to the ICU using mean NEWS scores by (**A)** sex (Female=0, Male=1); (**B)** by received vaccine (Unvaccinated=0, Vaccinated=1); (**C**) Comorbidity (Without comorbidity=0, With comorbidity=1).

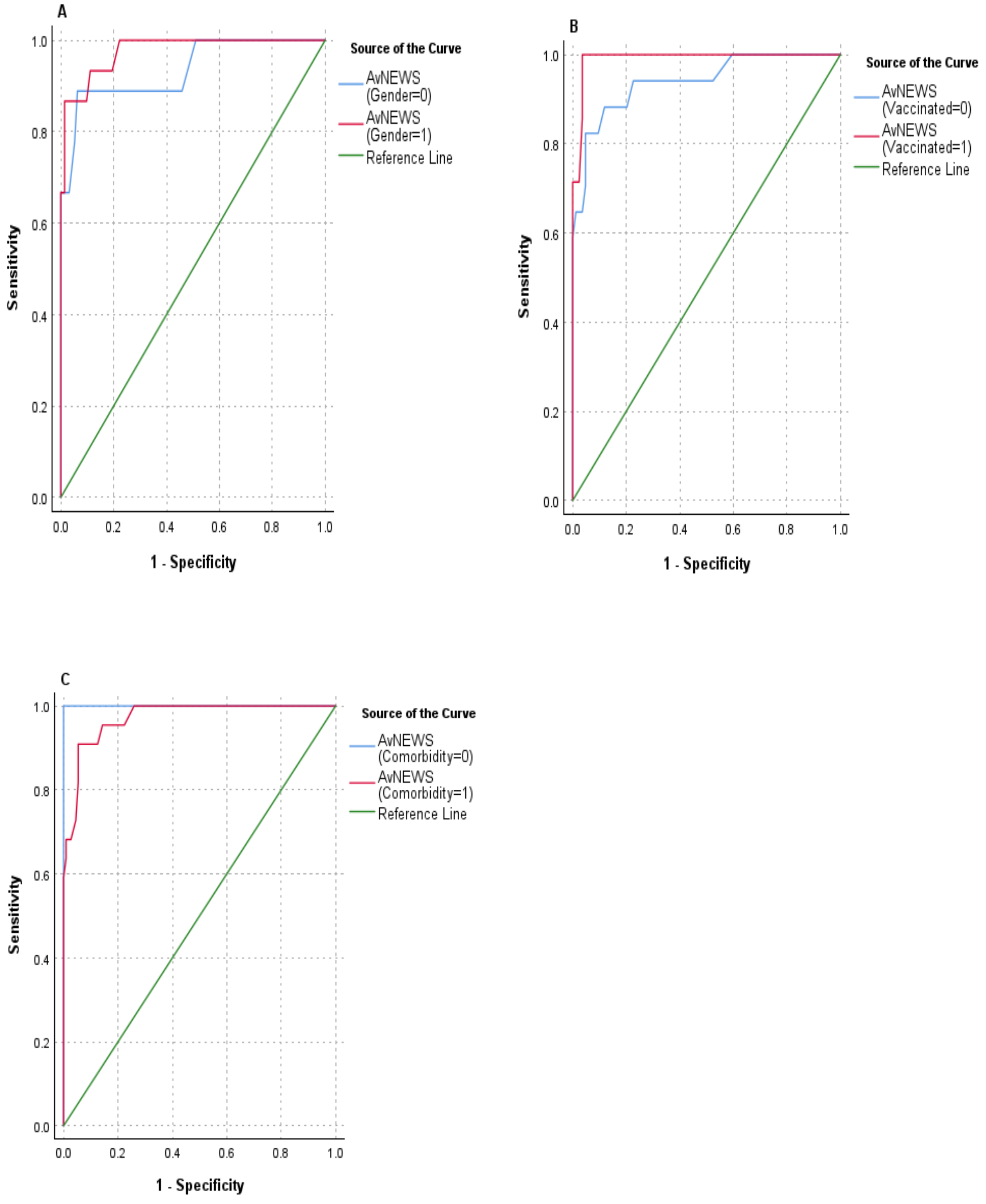

Figure 3S. ROC curve for patient's mortality using mean NEWS scores by (**A)** sex (Female=0, Male=1); (**B)** by received vaccine (Unvaccinated=0, Vaccinated=1); (**C**) Comorbidity (Without comorbidity=0, With comorbidity=1).
